# Acute Retinal Detachment Following Ozurdex Implant in a Nonvitrectomized Eye

**DOI:** 10.1155/crop/6671556

**Published:** 2026-03-01

**Authors:** Abdullah Alkhamees, Khaled Alsabah

**Affiliations:** ^1^ Department of Ophthalmology, Al-Bahar Eye Center, Ibn Sina Hospital, Kuwait City, Kuwait, ibnsinatrust.com

## Abstract

Intravitreal injection (IVI) of antivascular endothelial growth factors and dexamethasone implants are among the most performed procedures in ophthalmology, as they are used to treat various retinal diseases. Although considered safe, some rare but sight‐threatening complications can occur, like endophthalmitis and retinal detachment. Here, we report a case of a 53‐year‐old male with a history of lymphoma (treated with chemotherapy in 2016 and currently on Cellcept and low‐dose oral prednisolone), diabetes mellitus (DM), hypertension (HTN), and hyperlipidemia, who presented shortly after intravitreal Ozurdex injection in the left eye with retinal detachment (RD). Ozurdex is a valuable option for treatment of chronic diabetic macular edema (DME). This case highlights the need for a high index of suspicion for retinal detachment in pseudophakic patients with compromised retina, even after seemingly routine Ozurdex injection.

## 1. Introduction

Intravitreal injection (IVI) of antivascular endothelial growth factors (anti‐VEGF) and steroid implants like Ozurdex are among the most performed procedures in ophthalmology; they are used to treat many retinal diseases like diabetic retinopathy and retinal vascular occlusions. Although generally well tolerated, sight‐threatening complications can occur, including endophthalmitis and retinal detachment. The incidence of retinal detachment following IVI is approximately 0.033% [1, 2]. Other complications include intraocular pressure (IOP) elevation and implant migration into the anterior chamber.

We report the case of a 53‐year‐old male with multiple medical conditions including DM, HTN, hyperlipidemia, and lymphoma who is being treated with Ozurdex in both eyes for his DME and acutely developed retinal detachment after being injected in his left eye within 5 days. The patient received many IVI, including Ozurdex implant in both eyes in the past.

## 2. Case Presentation

We present a 53‐year‐old male with a history of Type 2 DM, HTN, and hyperlipidemia. He underwent chemotherapy in 2016 for the treatment of lymphoma and is currently on Cellcept 1‐g BID and oral prednisolone 5‐mg OD. He underwent phacoemulsification with intraocular lens implantation (IOL) in both eyes in 2017.

In late 2019, he was diagnosed with proliferative diabetic retinopathy (PDR) and received one IVI along with one session of panretinal photocoagulation (PRP) laser in both eyes. However, due to the COVID‐19 pandemic, he was unable to continue follow‐up. He was later referred to our hospital in February 2021, where he was diagnosed with regressed PDR and DME in both eyes. He was treated with IVI and subsequently switched to Ozurdex implants in both eyes.

**Figure 2:**
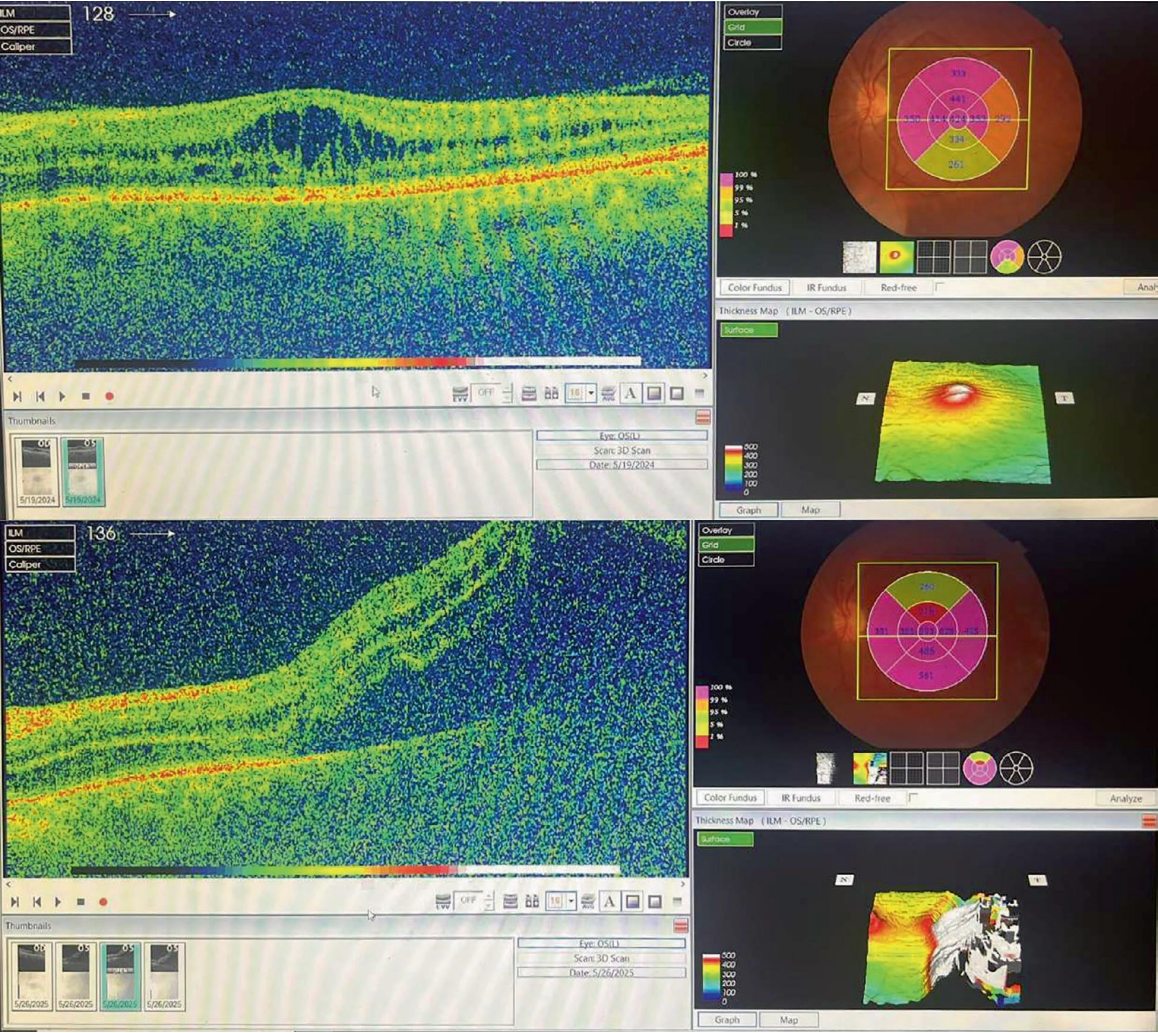
OCT scan of right eye top and left eye bottom at the day presented to ER showing right eye cystoid macular edema with partial PVD and left eye showing foveal‐involved retinal detachment.

The patient maintained regular follow‐up. In his recent visit, he received an Ozurdex implant in the right eye, followed by the left eye 2 days later, the two procedures were uneventful and was done by an experienced retina specialist. On the same day, the patient complained from flashes and floaters along with a black curtain in the nasal field. On Day 4 postinjection, he presented to our emergency department (ER). On examination, visual acuity in right eye was 20/70 and 20/150 in left eye. IOP was 14 mmHg in both eyes; anterior segment examination showed clear cornea and quiet anterior chamber with posterior chamber IOLs in both eyes. His fundus exam showed a flat retina with inferior laser marks and Grade 2 PVD in right eye, whereas the left eye showed retinal detachment from 11 till 5 o′clock along with inferior laser marks and no visible breaks were identified (Figure 1a,b). Optical coherence tomography (OCT) was done (Figure 2).

Figure 1(a) Ultrawide field fundus photo of the right eye showing incomplete inferior PRP laser scars. (b) Ultrawide field fundus photo of the left eye showing retinal detachment along with inferior incomplete PRP laser scars.

**Figure:**
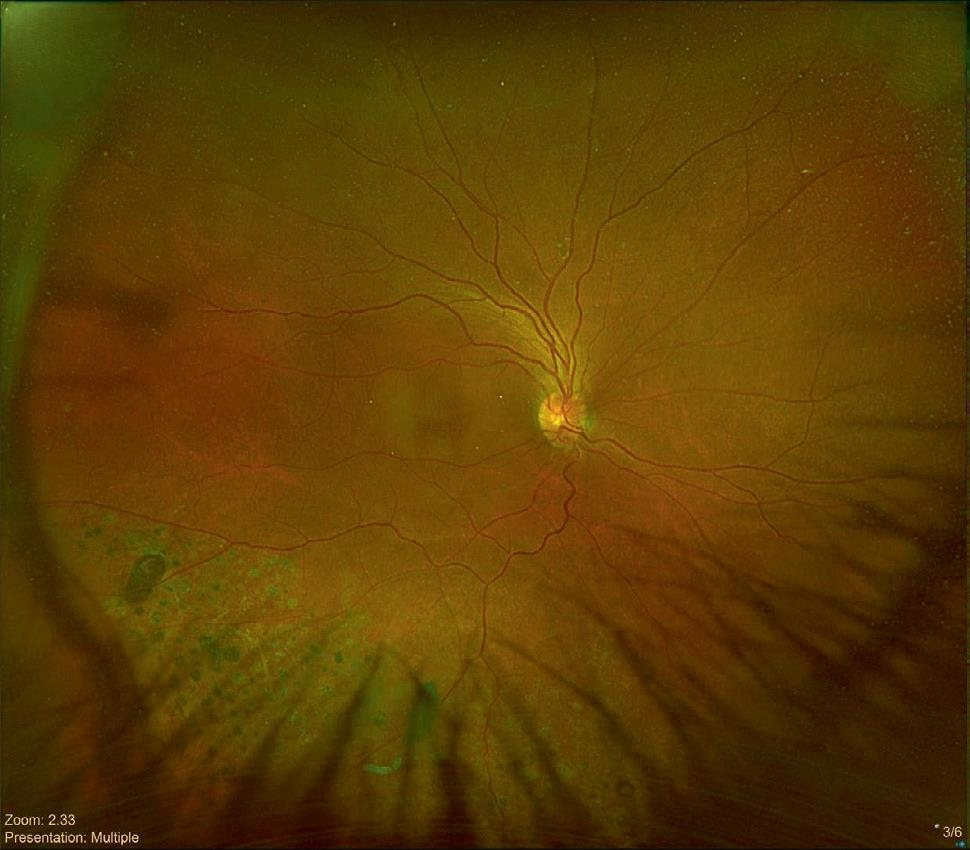
(a)

**Figure:**
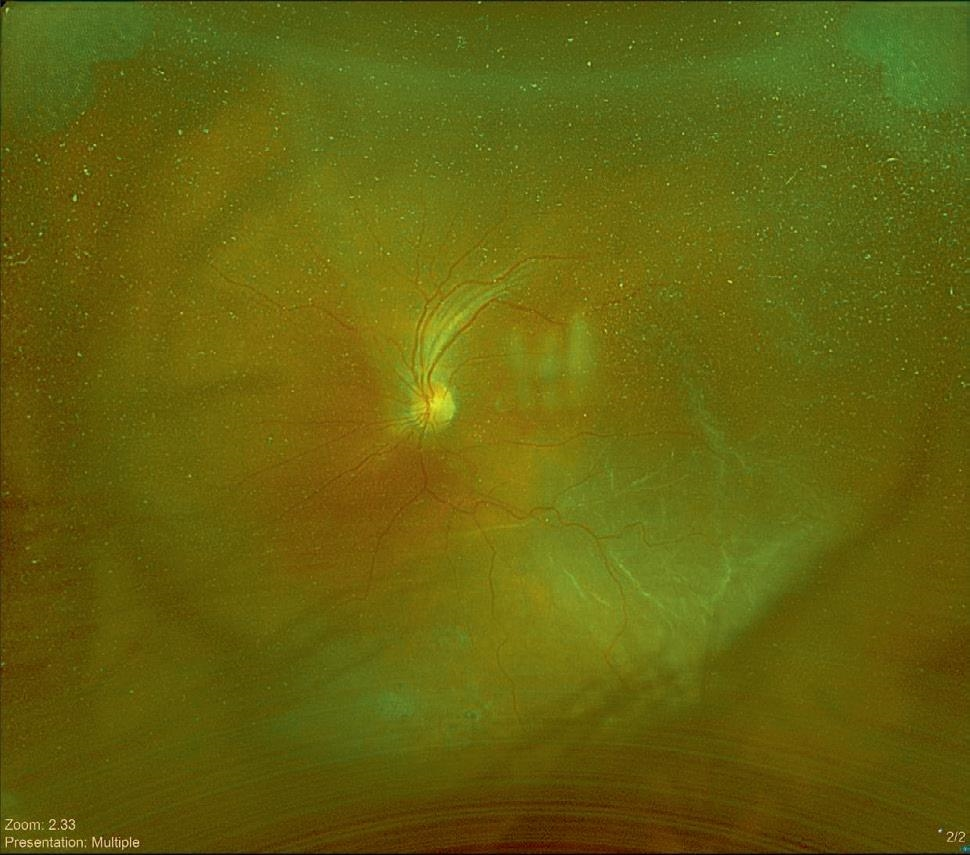
(b)

**Figure 3:**
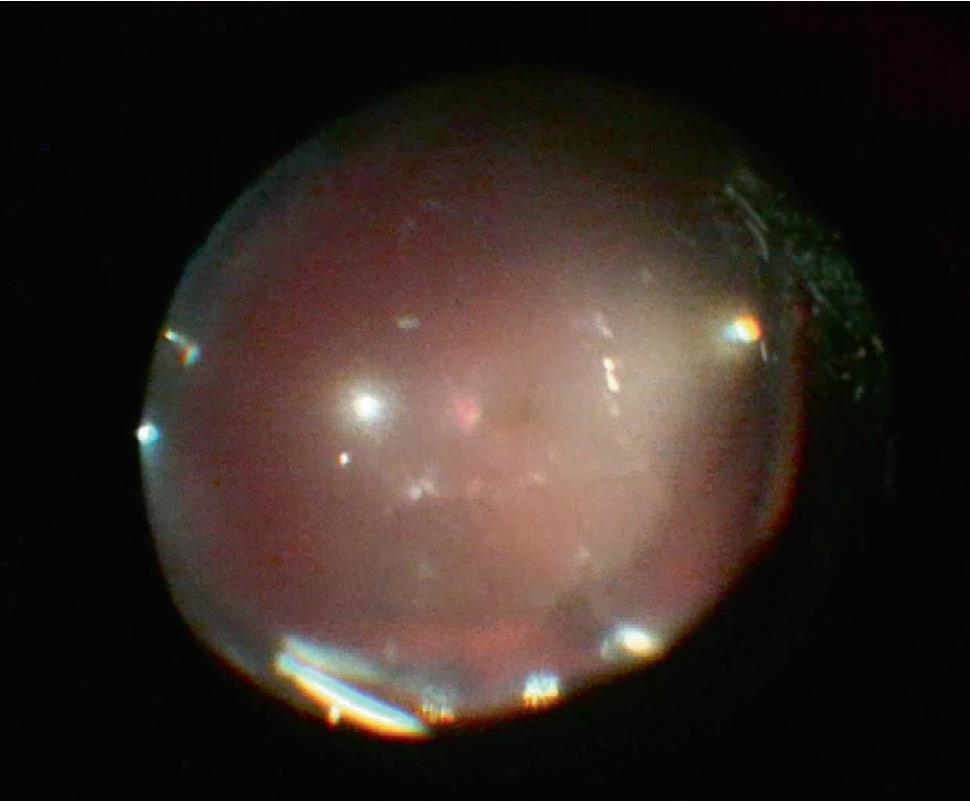
Intraoperative image after air–fluid exchange showing the implant inferiorly at the vitreous base.

The patient underwent PPV, SB, and gas tamponade (C_3_F_8_14%) the following day (Figure 3). The decision to do a combined PPV with a buckle was chosen because the initial examination and during the first surgery did not show any retinal breaks, and the buckle was placed to support the vitreous base and address any missed anterior small breaks.

## 3. Follow‐Up

**Figure 4:**
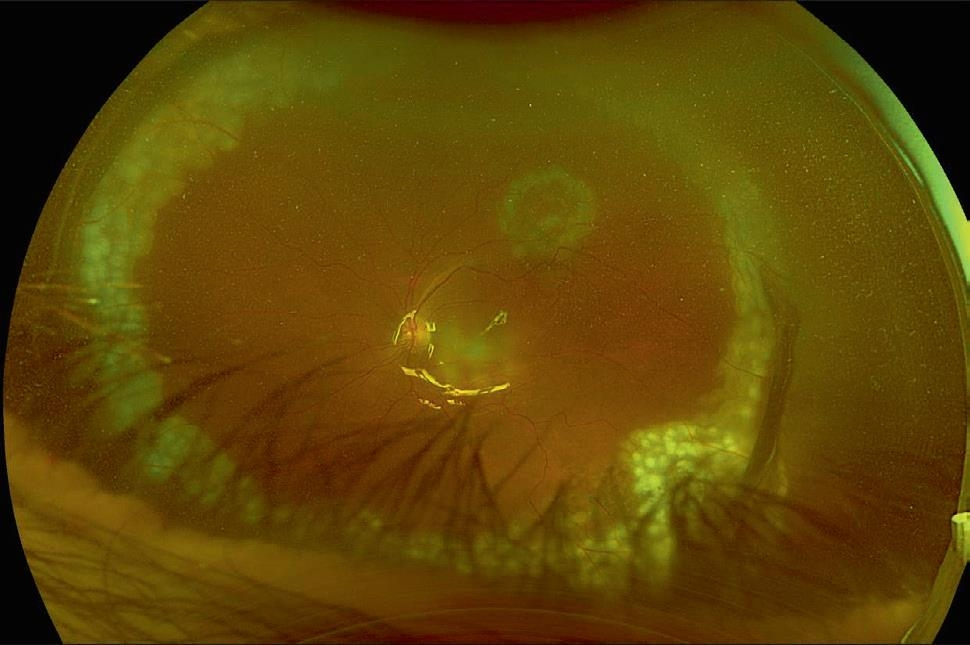
Postoperative wide field photo showing attached retina with silicone‐filled eye and laser retinopexy marks with the drainage retinotomy site at 1 o′clock from the first surgery.

Two weeks post initial surgery, fundus exam revealed some subretinal fluid inferiorly posterior to the buckle. Patient underwent reoperation. Intraoperatively, two retinal micro holes were identified at the 5 o′clock position posterior to the buckle. Subretinal fluid was drained, laser retinopexy applied, and silicone oil was instilled. Day 1 postoperative showed a reattached retina with adequate tamponade (Figure 4).

## 4. Discussion

In this discussion, we conducted a focused narrative review of the relevant literature to examine the potential mechanism and risk factors relevant to our case. Dexamethasone intravitreal implant (Ozurdex) is a widely used biodegradable corticosteroid indicated for the treatment of DME, retinal vein occlusion with macular edema, and noninfectious posterior uveitis. The implant is delivered through a 22‐gauge applicator, typically 4‐mm posterior to the limbus. Although considered safe and effective, known complications include elevated IOP, cataract, anterior migration of the implant in pseudophakic eyes, and rarely endophthalmitis. However, retinal detachment is an extremely rare event with a reported incidence of approximately 0.033% [1].

In our case, prior to the injection, the patient had regressed PDR along with incomplete PRP laser, chronic DME, and was under systemic immunosuppression treatment (Cellcept 2‐g BID and low‐dose prednisolone) due to history of lymphoma; he had multiple IVI in both eyes. The symptomatic onset just 1‐day postinjection strongly suggests the procedure acted as a direct mechanical trigger (patient was examined 2 days before the injection with dilated fundus exam and showed a flat retina). This could happen through a transient shift in vitreous pressure along with the sudden displacement during injection in a state of incomplete PVD, where persistent vitreoretinal adhesions remain despite surrounding vitreous separation. In such cases, the high velocity of the implant can lead to vigorous traction on the vitreous body; this indirect force is then transmitted to the retina and in patients with preexisting firm vitreoretinal adhesions can cause traction [3]. The force from the injection can create a sudden displacement of the vitreous gel, transmitting acute tractional forces to these points of abnormal adhesions, causing a retinal tear. The patient′s underlying medical history amplified this risk. Several factors likely contributed to a compromised retinal state, including diabetes and PDR, where diabetic eyes often exhibit anomalous PVD along with vitreoschisis, as well as possible alterations in the vitreoretinal interface that may develop after Ozurdex treatment. This makes the retina more susceptible to tractional forces [4–6]. Prior phacoemulsification is also a well‐established risk factor for subsequent RD as it can alter and destabilize the vitreous, predisposing the eye to traction‐induced breaks [7].

A critical aspect of this case was the initial inability to identify a retinal break, both pre‐operative and during the first surgery. The microholes were only discovered during the reoperation at the 5 o′clock position. This location is consistent with the injection site which may raise the possibility of a direct iatrogenic break from the applicator needle. However, given the preexisting risk profile, it is likely that these were small traction‐induced holes that were difficult to visualize initially, perhaps obscured by peripheral laser scars.

In conclusion, this unique case represents a rare incidence of retinal detachment in a non vitrectomized eye following Ozurdex injection, as it occurred in an acute setting along with the patient’s multiple risk factors; it also describes the difficulty and challenge in identifying microholes requiring reoperation. Ophthalmologists should maintain a high index of suspicion for early symptoms of RD following IVI and consider extended observation postinjection in high‐risk eyes.

**Nomenclature**
IVI: intravitreal injection
PPV: pars plana vitrectomy
SB: scleral buckle
PVD: posterior vitreous detachment
VEGF: vascular endothelial growth factor
IOP: intraocular pressure
DM: diabetes mellitus
HTN: hypertension
DME: diabetic macular edema
IOL: intraocular lens
PRP: panretinal photocoagulation
PDR: proliferative diabetic retinopathy
ER: emergency department
OCT: optical coherence tomography

## Funding

No funding was received for this manuscript.

## Consent

Written informed consent for publication was taken from the patient.

## Conflicts of Interest

The authors declare no conflict of interest.

## Data Availability

Data sharing is not applicable to this article as no datasets were generated or analyzed during the current study.

## References

[1] Brown K. R. , Yannuzzi N. A. , Smiddy W. E. , Gregori N. Z. , Berrocal A. M. , Haddock L. J. , Albini T. A. , and Flynn H. W. , Rhegmatogenous Retinal Detachment After Intravitreal Injection, Ophthalmol Retina.(2021) 5, no. 2, 178–183, 10.1016/j.oret.2020.07.007. 32673672

[2] Celik N. , Khoramnia R. , Auffarth G. U. , Sel S. , and Mayer C. S. , Clinical Characteristics and Outcome of Posterior Cystoid Macular Degeneration in Chronic Central Serous Chorioretinopathy, Retina. (2020) 40, no. 9, 1742–1750, 10.1097/IAE.0000000000002683, 31815880. 31815880 PMC7447130

[3] Meyer C. H. , Klein A. , Alten F. , Liu Z. , Stanzel B. V. , Helb H. M. , and Brinkmann C. K. , Release and Velocity of Micronized Dexamethasone Implants With an Intravitreal Drug Delivery System: Kinematic Analysis With a High-Speed Camera, Retina. (2012) 32, no. 10, 2133–2140, 10.1097/IAE.0b013e31825699e5, 2-s2.0-84871303992, 23060033. 23060033

[4] Sebag J. , Anomalous Posterior Vitreous Detachment: A Unifying Concept in vitreo-retinal Disease, Graefe′s Archive For Clinical and Experimental Ophthalmology. (2004) 242, no. 8, 690–698, 10.1007/s00417-004-0980-1, 2-s2.0-4544323408, 15309558. 15309558

[5] Bakri S. J. and Omar A. , Evolution of Vitreomacular Traction Following the Use of the Dexamethasone Intravitreal Implant (Ozurdex) in the Treatment of Macular Edema Secondary to Central Retinal Vein Occlusion, Journal of Ocular Pharmacology and Therapeutics. (2012) 28, no. 5, 547–549, 10.1089/jop.2011.0184, 2-s2.0-84866985056, 22537269. 22537269

[6] Alpay A. , Posterior Vitreous Detachment Rate Following Intravitreal Dexamethasone Injection, International Journal of Ophthalmology. (2019) 12, no. 8, 1298–1303, 10.18240/ijo.2019.08.10, 2-s2.0-85074100413, 31456920. 31456920 PMC6694056

[7] Mitry D. , Charteris D. G. , Fleck B. W. , Campbell H. , and Singh J. , The Epidemiology Of Rhegmatogenous Retinal Detachment: Geographical Variation and Clinical Associations, British Journal of Ophthalmology. (2010) 94, no. 6, 678–684, 10.1136/bjo.2009.157727, 2-s2.0-77953786468, 19515646. 19515646

